# Assessment of the effectiveness of a course in major chemical incidents for front line health care providers: a pilot study from Saudi Arabia

**DOI:** 10.1186/s12909-022-03427-2

**Published:** 2022-05-09

**Authors:** Nidaa Bajow, Shahnaz Alkhalil, Nisreen Maghraby, Saleh Alesa, Amal Al Najjar, Samer Aloraifi

**Affiliations:** 1 Security Forces Hospital Program, P O Box 89489, Riyadh, 11682 Kingdom of Saudi Arabia; 2 Faculty of Engineering and Technology, Alzaytoonah University, Amman, Jordan; 3 King Fahad University Hospital Collage of Medicine, Imam Abdulrahman Bin Faisal University, Dammam, Kingdom of Saudi Arabia; 4 General Directorate of Medical Services Special Security Forces, Riyadh, Kingdom of Saudi Arabia; 5 Hail Health Cluster Ministry of Health, Hail, Kingdom of Saudi Arabia

**Keywords:** Disaster medicine education, Decontamination, Major chemical incident, Mass chemical exposure, Personal protective equipment, Competency based, Simulation enhanced training

## Abstract

**Background:**

Mass chemical exposure emergencies are infrequent but can cause injury, illness, or loss of life for large numbers of victims. These emergencies can stretch and challenge the available resources of healthcare systems within the community. Political unrest in the Middle East, including chemical terrorist attacks against civilians in Syria and increasing chemical industry accidents, have highlighted the lack of hospital preparedness for chemical incidents in the region. This study aimed to evaluate the effectiveness of a course designed to empower frontline healthcare providers involved in mass casualty incidents with the basic knowledge and essential operational skills for mass chemical exposure incidents in Saudi Arabia.

**Methods:**

A mixed-methods approach was used to develop a blended learning, simulation enhanced, competency-based course for major chemical incidents for front line healthcare providers. The course was designed by experts from different disciplines (disaster medicine, poisoning / toxicology, and Hazard Material Threat - HAZMAT team) in four stages. The course was piloted over five days at the Officers Club of the Ministry of Interior (Riyadh, Saudi Arabia). The 41 participants were from different government health discipline sectors in the country. Pre- and post-tests were used to assess learner knowledge while debriefing sessions after the decontamination triage session and simulation-enhanced exercises were used for team performance assessment.

**Results:**

The overall knowledge scores were significantly higher in the post-test (69.47%) than the pre-test (46.3%). All four knowledge domains also had significant differences between pre- and post-test results. There were no differences in the pre and post-test scores for healthcare providers from the different health disciplines. A one-year post-event survey demonstrated that participants were satisfied with their knowledge retention. Interestingly, 38.3% had the opportunity to put this knowledge into practice in relation to mass chemical exposure incidents.

**Conclusion:**

Delivering a foundation level competency-based blended learning course with enhanced simulation training in major chemical incidents for front line healthcare providers may improve their knowledge and skills in response to such incidents. This in turn can improve the level of national preparedness and staff availability and make a crucial difference in reducing the health impacts among victims.

**Supplementary Information:**

The online version contains supplementary material available at 10.1186/s12909-022-03427-2.

## Background

A large-scale toxic chemical release in an inhabited area has a high likelihood of leading to a mass chemical exposure incident, which could subsequently have a significant impact on the healthcare system [1, 2]. While these mass chemical exposure emergencies are infrequent, they have the potential to cause injury, illness, or loss of life for many victims that can affect, stretch and challenge the available resources in the healthcare system [3, 4].

Acute chemical exposure may occur in a wide range of events such as unintentional release (e.g. industry or chemical transportation accidents) or terrorist acts [5–7]. Many factors can impact healthcare providers response to Hazardous Materials (HAZMAT) and /or Chemical Biological Radiological and Nuclear (CBRN) threats, thereby complicating a straightforward emergency response, such as the “fear factor,” access to appropriate education or training, and insufficient knowledge or awareness of response to CBRN events [8–13].

Political unrest in the Middle East and the occurrence of chemical terrorist attacks against civilians in Syria between March 2013–March 2017 have highlighted the lack of hospital preparedness for chemical incidents in the region. The growing risks of chemical industry accidents due to suboptimal industry environments for workers in Saudi Arabia also raise the need for education and training of frontline healthcare providers to prepare for mass chemical exposure incidents [14–20]. The suboptimal preparedness of Saudi hospitals for disaster health, specifically CBRN threats, are attributed to inadequate education, training and lack of exercises that promote disaster readiness [21–23].

Experience with mass chemical exposure incidents suggests that first responders and emergency department receivers should know the basic characteristics of chemical agent toxidromes, identification and recognition of the hazards, required mitigation measures for safety and protection in addition to integration and coordination with involved organizations [24–27].

Competency-based education and training are the key element for disaster health preparedness [28, 29]. Training can increase staff self confidence that is important for improving the level of preparedness and, subsequently, staff availability [30]. Multidisciplinary training and coordination should be integrated within the healthcare system as part of disaster health planning aimed at accurate detection, surveillance and emergency response [31]. Previous studies in Saudi Arabia demonstrated the need for a competencies-based multidisciplinary training program which included all hazards threatening the country as part of disaster mitigation measures [14, 32, 33]. The escalating incidence of intentional and non-intentional major chemical risks and inadequate healthcare system preparedness in the Middle East in general, and Saudi Arabia specifically, highlight the need for competency-based major chemical incident education and training programs [16–23, 34].

The main aim of this study is to evaluate a course designed to provide front line healthcare providers who are likely to be involved in major incidents and disasters with the basic knowledge and essential operational skills for mass chemical exposure incidents.

## Methods

### Course overview

A mixed-methods approach was used to develop a blended-learning, simulation enhanced, and competency-based course in major chemical incidents for healthcare providers. This 37-hour (five day) course was developed in response to an existing need [27]. The course was piloted on 18 March 2018 at the Officer’s Club of the Ministry of Interior, Riyadh, Saudi Arabia. The course was prepared in English by seven experts in disaster medicine with subject matter experts from toxicology and the civil defence HAZMAT team following a four-stage approach. During the first 3 days, interactive lectures were introduced on all four theoretical and practical knowledge domains. On the fourth day, four morning practical sessions were conducted on decontamination facilities and equipment, donning and doffing PPE (Personal Protective Equipment), decontamination processes and triage. A tabletop exercise for prehospital chemical incident response was conducted in the afternoon session. On the last day, a functional ‘putting it all together’ exercise was conducted for self-evacuation of patients after an industry incident to a hospital emergency department.

The four stages of the course development, details of the knowledge domains involved, and the practical components taught are presented in detail in the online Additional Materials and briefly summarised below.

#### Stages of course development

##### Stage 1: identify need for competency-based course in CBRN threats in Saudi Arabia

The first stage of the course development involved a literature review of currently available courses and information to determine the need for a competency-based course in disaster health including CBRN subjects in Saudi Arabia.

##### Stage 2: preference for major chemical incident course vs. other unconventional threats (biological, nuclear, radiological threats)

The subject matter experts used multiple brainstorming sessions to identify which CBRN threats were more likely in the Middle East region and analysed the characteristics of the possible CBRN threats that could specifically affect the Saudi community [27]. After reviewing all the likely threats and hospital preparedness levels, the experts identified chemical threats as the most important and likely threats in Saudi Arabia.

##### Stage 3: delineation of a course aimed at the foundation level for chemical major incidents

Most Disaster Health Education frameworks are designed according to international standards and local context and are composed of different levels with associated competencies [14, 35].Experts recognized that it would be unfeasible to provide all healthcare providers (responders and receivers) with advanced level CBRN training and agreed that all front line healthcare providers should receive at least foundation level training for CBRN threats [27, 36, 37]. This level allows healthcare workers to do initial assessments, identify the chemical hazards, assist the HAZMAT team in warm zones with documentation and registration, ambulatory decontamination, medical triage post decontamination, and exchange information with the poison centers [27, 37]. (see Table AM1 in Additional Materials file 2).

##### Stage 4: competencies development

The core competencies set for CBRN threats, including mass chemical exposure education, were identified after a comprehensive literature review. Four domains were determined according to participants’ roles and tasks and six levels of proficiency (Bloom’s Education Taxonomy) were used to establish the core competencies and sub-competencies for the foundation level in major chemical incidents (see Table A1 in Additional Materials file 1) [27, 38–41].

### Teaching strategy

A blended learning technique was used combining interactive presentations, tabletop exercises, drills, and experiential/ hands-on exercises to deliver knowledge and skills in the four domains and promote higher cognitive engagement [14, 32] The theoretical content was presented over the first 3 days of the course, followed by the practical components on days 4 and 5.

#### The four Main theoretical domains

##### Threat identification domain

The threat identification domain was composed of one core competency and 6 sub-competencies (see Table A1 in Additional Material file 1). The domain includes three main topic areas: (i) different chemical agents’ experiences and scenarios from the past, (ii) factors influencing the effects of chemical incidents, and (iii) the role of the poisoning centre during a major chemical incident. See Additional Material file 1 for more details on the sub-topics and teaching methods involved for this domain.

##### Health effect of chemical agent domain

The health effect of the chemical agent domain was composed of one core competency and five sub-competencies (see Table A1 in Additional Material file 1). This domain describes the potential health impacts for suspected chemical hazards and its health consequences. See Additional Material for more details on the sub-topics and teaching methods involved for this domain.

##### Response to major chemical incident domain

The response to the major chemical incident domain was composed of one core competency with five sub-competencies for pre-hospital response, six sub-competencies for hospital response and one sub-competency for toxic trauma treatment (see Table A1 in Additional Material file 1). This domain included information on key activities involved in the response phase such as cooperation and communication between different organizations, scene management, site access and triage. The content also included information on hospital responses and life-saving treatments. See Additional Material for more details on the sub-topics and teaching methods involved for this domain.

##### The basic concept in protection and safety domain

The basic concept in the protection and safety domain is composed of one core competency and seven sub-competencies (see Table A1 in Additional Material file 1). The rationale, function, type, limitation of personal protective equipment (PPE) and decontamination processes were presented in this domain. See Additional Material for more details on the sub-topics and teaching methods involved for this domain.

#### Practical components of the course

On days 4 and 5, small group sessions and simulation-based exercises were conducted. The sessions were composed of an awareness section and hands-on sections for emergency response skills that were presented in the theory section. More details on sub-topics and teaching methods involved for each of the exercises are presented in the Additional Materials.

The awareness session introduced the HAZMAT detection and management vehicle, the chemical decontamination tent, the decontamination car, and the types of personal protective equipment.

The hands-on sessions focused on skills training for different operational aspects as follows:

- Personal protective equipment (PPE) Level C technique for donning and doffing.
- Ambulatory and non-ambulatory decontamination including wound decontamination in different areas, e.g. eye, chest and arm. For the PPE Level C technique and decontamination sessions, errors in skill performance were corrected immediately and then repeated to achieve the skills at the required level.
- Triage decontamination using 10 prepared cases with clinical information printed on a white card for an organophosphate agent. Each group was asked to follow the flow chart provided to categorize each patient and identify their priority for decontamination. After 15 minutes the instructor discussed the triage category for each patient and provided explanations for any incorrect categorizations.

In the afternoon session on day 4, a tabletop simulation exercise involving a nerve agent released in the Annual Conference for Police Academies that resulted in 30 injuries was conducted. On day 5, a drill for self-evacuation victims of an explosion of ethylene oxide storage tanks in a factory to a hospital emergency department was conducted. Twelve standardized patients were used in this scenario and two-way radios were used to facilitate communication between the players with the regional command centre and toxicology centre.

These sessions were preceded by a briefing period, during which participants psychological safety was ensured. This included clearly stating that it was a safe simulated learning environment where mistakes can happen without endangering anyone.

#### Instructors

There were 14 instructors in total. Five were from the Toxicology Centre and Emergency Department of King Fahad Medical Centre. The others were as follows; two from the Ministry of Interior (Disaster Medicine Unit and Emergency Department), one from the Emergency Department of King Saud Medical City Centre, three from the Occupational Safety and Health Administration (OSHA) team at King Faisal Specialist Centre and three from the Civil Defence HAZMAT team. Of these instructors, 11 were medical instructors specialized in disaster medicine, toxicology and poisoning. Three non-medical instructors were from the HAZMAT team, experts based in the management of hazardous substance unit - civil defence.

#### Course participants

Forty-one health care providers were enrolled from the main government health sectors across Saudi Arabia (i.e. hospitals, polyclinics and emergency medical services). The learners had previously attended awareness or /basic knowledge courses in disaster health management over the last 2 years and were likely to be involved in a major chemical incident response. All participants consented to participation before the course, and the study was approved by the Institutional Review Board of Princess Nourah Bint Abdulrahman University (IRB log NO. 21-0202E).

#### Outcome measures

Three stages of Kirkpatrick’s Four Model were used to develop cognitive assessment, feedback evaluation and post event questionnaire forms. Details of these measures are presented in the Additional Materials file 1, Table A2 and briefly summarised below.

##### Cognitive assessment level 2 (knowledge, skills & attitude)

To test theoretical knowledge, learners completed a 25 multiple-choice question examination before and after the course. Content and difficulty were same for all pre-course and post-course assessments. Individual skills assessment was based on immediate correction of mistakes during practice with skills repeated in decontamination, donning, and doffing of the PPE - Level C. Team performance in the decontamination triage session and simulation-based exercise (tabletop exercise and drill) was assessed using guided instructor observations. The “Role Evaluation Checklist” was used by the instructor as a guide for their observations. The checklist identifies specific tasks and the instructor determined if competency was achieved, not achieved or not observed followed by comments and recommendations. In addition, observers used timed “Data observation forms” to log the sequence of events during the functional drill.

These were used later during the debriefing sessions that were led by the Master Exercise Controller forming a base for self-evaluation of the actions and measures that were taken for response management**.**

##### Feedback (learner satisfaction)

An 11-item evaluation questionnaire was used to obtain participant feedback at the end of the course regarding the effectiveness of the course and suggestions on how the course might be improved.

##### Measurement of behaviour level post-course

One year after the course, a follow-up survey was sent to the participants by email. Those who did not respond to the email were contacted by phone whenever possible. The questions mainly included whether the participant had applied the course principles, obtained the knowledge needed or participated in a major chemical incident post-course. Additional Materials file 1, Table A2.

#### Statistical data analysis

Comparisons between pre- and post-test scores for different major chemical incident domains and the overall score were made using descriptive analyses and non-parametric Wilcoxon signed rank test. A Kruskal Wallis test was used to compare the scores by profession. Descriptive analyses were used to examine participant’s evaluations of the course effectiveness. All data were analysed using Statistical Package for Social Science (IBM-SPSS; Statistics) version 26 for Windows and the level of statistical significance was set at *P* ≤ 0.05. The reliability of the knowledge and practice items were analysed using Cronbach’s alpha.

## Results

### Participant knowledge

Assessment of the pre- and post-test score reliability for the knowledge items in the course produced a Cronbach’s alpha coefficient of stability of 0.803 indicating acceptable reliability.

The study recruited 41 participants (36 males; 5 females), but two did not do the pre-test and five did not do the post-test. Of the 34 who took both tests, there were 22 paramedics (64.7%), 6 physicians (17.6%), 4 nurses (11.7%), 1 radiology technician (2.9%) and 1 pharmacist (2.9%). The overall score on the pre-test was 46.3% which increased to 69.47% on the post-test.

Domain 1 evaluated Threat identification, Domain 2 evaluated Health effects of chemical agent, and Domain 3 evaluated Response to major chemical incidents while Domain 4 was concerned with the Basic concepts in protection and safety. As seen in Table 1, all domains showed increased correct responses by the participants after the training session. All individual questions, except for questions 13 and 24 (lower) and question 20 (no change), showed an increase in score. Wilcoxon signed rank tests demonstrated that the post-test scores for all four domains were significantly higher than the pre-test scores (Table 2).

**Table 1:** Distribution of correct responses to the questionnaire items before (pre) and after (post) training (*n* = 34)

		Pre-test		Post-test		
Items	Questionnaire items	No.	%	No.	%	Improvement
1	One of the following is an example of chemical removal of contaminant	18	46.15%	31	86.11%	72.22%
2	Checklist of communication with clinical toxicology include:	31	79.49%	33	91.67%	6.45%
3	Which statement is true regarding the reactivity of hazardous materials with water?	6	15.38%	16	44.44%	166.67%
4	During a HAZMAT incident, when do patients need to be decontaminated?	11	28.21%	13	36.11%	18.18%
5	Persistence of Chemical agent cause effects long after release. Which of these agents has the most persistency:	3	7.69%	24	66.67%	700.00%
6	Nerve agent poisoning may be rapidly fatal. Beside appropriate decontamination, which of the following should be administered?	6	15.38%	19	52.78%	216.67%
7	All of the following agents are blistering agents except?	16	41.03%	30	83.33%	87.50%
8	Which the following clinical effects would be least likely to occur from exposure from sulfur mustard?	14	35.90%	14	38.89%	0.00%
9	What is the primary objective at any hazardous materials` release?	18	46.15%	24	66.67%	33.33%
10	Which of the following statements about a spill in which wind is blowing from north is correct?	8	20.51%	23	63.89%	187.50%
11	When you should call the Poison Control Center:	8	20.51%	14	38.89%	75.00%
12	Regarding Chemical Protective clothing which of the following is incorrect:	12	30.77%	14	38.89%	16.67%
13	The Clinical Toxicologist of the 24 h information /consultation services of the PCC will provide the specialist on duty with further instructions and advice about the following, all are true except one:	9	23.08%	2	5.56%	−77.78%
14	Which the following chemical agents caused the greatest number of fatalities in World war1?	7	17.95%	30	83.33%	328.57%
15	During the decontamination of critical ill victims, which body area requires particular attention?	20	51.28%	22	61.11%	10.00%
16	What are the roles of Poison Control Center in CWI?	29	74.36%	32	88.89%	10.34%
17	Which of the following is not a source of secondary contamination (cross-contamination);	10	25.64%	20	55.56%	100.00%
18	Mustard is considered a:	7	17.95%	26	72.22%	271.43%
19	A 37-year-old man arrives at the emergency department (ED) after exposure to an organophosphate. He is severely symptomatic, and atropine is given. When should atropine treatment be discontinued?	7	17.95%	16	44.44%	128.57%
20	In a simple Asphyxiant agent. What main organ system is affected?	25	64.10%	25	69.44%	0.00%
21	You suspect a patient has a cholinergic syndrome. Which of the following, if found, would make you question your diagnosis?	14	35.90%	17	47.22%	21.43%
22	Which the following chemical warfare agents is the most potent?	8	20.51%	24	66.67%	200.00%
23	Which the following HAZMAT scene control zones requires the highest level of personal protective equipment (PPE)?	12	30.77%	30	83.33%	150.00%
24	Antidote type, dose, and preparation should be known by:	26	66.67%	25	69.44%	−3.85%
25	Before at least primary decontamination, the most urgent lifesaving procedure/s that should be carried out is:	13	33.33%	16	44.44%	23.08%

**Table 2:** Pre- and Post-Test Knowledge about Major Chemical Incidents

Domains	Mean	SD	Wilcoxon Signed Ranks Test (Z)	***P*** Value
**Pre-test Domain 1 score**	2.3529	0.84861	−4.739	0.000002^a^
**Post-test Domain 1 score**	4.3235	1.00666		
**Pre-test Domain 2 score**	2.6765	1.36450	−4.507	0.000007 ^a^
**Post-test Domain 2 score**	4.8824	1.85480		
**Pre-test Domain 3 score**	3.2059	1.24996	−3.801	0.000144 ^a^
**Post-test Domain 3 score**	4.5882	1.41673		
**Pre-test Domain 4 score**	2.1765	1.26660	−2.868	0.004125 ^a^
**Post-test Domain 4 score**	2.9706	.96876		
**Overall Pre-test score**	10.4118	3.06612	−4.891	0.000001 ^a^
**Overall Post-test score**	16.7647	3.79840		

^a^: Statistically significant (*P* ≤ 0.05)

Key terms: Domain1(Threat Identification), Domain2(Health Effect of Chemical Agent),Domain3 (Response to Major Chemical Incident), Domain 4(The Basic Concepts in Protection and Safety)

As the participants were from different healthcare specialties (i.e., physicians, paramedics, nurses, technicians, and pharmacists), the pre- and post-test scores for each group were compared by Kruskal Wallis H Test. In the pre-test, nurses scored higher (mean score ± standard deviation and mean ranks) in domain 1 (6.25 ± 1.71, 20.00) and domain 4 (12.25 ± 3.86, 20.38), compared to other health professionals but this difference was not statistically significant (*p* > 0.005). In the post-test, the radiology technician scored higher (mean score ± standard deviation and mean ranks) in domain 1 (9.00 ± 00, 25.50) and domain 2 (5.00 ± 0.00, 24.00) compared to other health professionals but again this difference was not significant (*p* > 0.005), as shown in Table 3.

**Table 3:** Comparison of pre-test and post-test score among participants from different groups (*n* = 34)

Domains	Position	N	Mean	SD	Mean Rank	KW-H	df	P
**Pretest score (Domain 1)**	**Nurse**	4	6.25	1.71	20.00	3.479	4	0.481
	**Paramedic**	22	4.32	1.55	16.61			
	**Radiology technician**	1	3.00	0.0	33.00			
	**Physician**	6	4.50	2.07	17.17			
	**Pharmacist**	1	4.00	0.0	13.50			
	**Total**	34	4.53	1.71				
**Pretest score (Domain 2)**	**Nurse**	4	3.25	1.26	26.13	7.774	4	0.100
	**Paramedic**	22	2.55	1.10	14.50			
	**Radiology technician**	1	2.00	0.0	30.50			
	**Physician**	6	2.17	0.98	19.58			
	**Pharmacist**	1	5.00	0.0	23.50			
	**Total**	34	2.62	1.16				
**Pretest score (Domain 3)**	**Nurse**	4	3.25	2.22	22.38	7.372	4	0.117
	**Paramedic**	22	2.27	1.24	14.43			
	**Radiology technician**	1	5.00	0.0	18.00			
	**Physician**	6	2.67	1.03	25.33			
	**Pharmacist**	1	5.00	0.0	18.00			
	**Total**	34	2.62	1.44				
**Pretest score (Domain 4)**	**Nurse**	4	12.25	3.86	20.38	5.455	4	0.244
	**Paramedic**	22	8.95	3.18	18.57			
	**Radiology technician**	1	10.00	0.0	2.00			
	**Physician**	6	10.17	3.43	12.83			
	**Pharmacist**	1	11.00	0.0	26.00			
	**Total**	34	9.65	3.29				
**Overall Pretest score**	**Nurse**	4	10.00	4.08	23.50	4.790	4	0.310
	**Paramedic**	22	9.14	2.78	15.18			
	**Radiology technician**	1	14.00	0.0	27.00			
	**Physician**	6	9.33	2.73	18.83			
	**Pharmacist**	1	10.00	0.0	27.00			
	**Total**	34	9.44	2.88				
**Posttest score (Domain 1)**	**Nurse**	4	11.25	3.20	22.00	4.297	4	0.367
	**Paramedic**	22	7.00	3.41	15.30			
	**Radiology technician**	1	9.00	0.0	25.50			
	**Physician**	6	6.67	3.78	19.92			
	**Pharmacist**	1	10.00	0.0	25.50			
	**Total**	34	7.59	3.57				
**Posttest score (Domain 2)**	**Nurse**	4	6.25	2.63	18.63	2.868	4	0.580
	**Paramedic**	22	3.23	1.60	17.25			
	**Radiology technician**	1	5.00	0.0	24.00			
	**Physician**	6	2.67	1.97	14.42			
	**Pharmacist**	1	10.00	0.0	30.50			
	**Total**	34	7.59	3.57				
**Posttest score (Domain 3)**	**Nurse**	4	4.50	1.73	22.50	1.841	4	0.765
	**Paramedic**	22	3.32	1.52	17.07			
	**Radiology technician**	1	5.00	0.0	20.50			
	**Physician**	6	2.83	1.60	14.75			
	**Pharmacist**	1	3.00	0.0	20.50			
	**Total**	34	3.41	1.56				
**Posttest score (Domain 4)**	**Nurse**	4	16.75	5.97	17.75	3.635	4	0.458
	**Paramedic**	22	12.45	6.84	17.48			
	**Radiology technician**	1	20.00	0.0	28.50			
	**Physician**	6	16.00	3.10	13.75			
	**Pharmacist**	1	19.00	0.0	28.50			
	**Total**	34	14.00	6.29				
**Overall Posttest score**	**Nurse**	4	15.50	7.19	23.50	6.405	4	0.171
	**Paramedic**	22	13.55	5.83	15.93			
	**Radiology technician**	1	19.00	0.0	30.00			
	**Physician**	6	12.17	6.31	14.67			
	**Pharmacist**	1	18.00	0.0	32.50			
	**Total**	34	13.82	5.88				

KW-H=Kruskal Wallis H Test

Key terms: Domain1(Threat Identification), Domain2(Health Effect of Chemical Agent),Domain3 (Response to Major Chemical Incident), Domain 4(The Basic Concepts in Protection and Safety)

The knowledge and skills gained from the theoretical and hands-on sessions were obvious during the debriefing sessions for both the functional and tabletop exercise. The main objectives and tasks were generally achieved. In the tabletop exercise, team performance regarding scene coordination with civil defence and the poison centre and decontamination triage were the best met objectives. During the functional exercise, the activation of the emergency plan and the decontamination process were the best met objectives.

### Learner feedback

Only 27 participants (79.4%) answered the post-course evaluation survey as shown in Table 4 with all of them indicating they were personally interested in the course and had met their personal goals.

**Table 4:** Overall participants post-evaluation survey of the course in major chemical incidents piloted in Saudi Arabia, 2018

Personally Interested (Yes/ No)	100% Yes					
Personal Goals Met (Yes/ No)	100% Yes					
Statement	Strongly Agree 1	Agree 2	Neutral 3	Disagree 4	Strongly Disagree 5	Positive Answers*
1. Overall, the pre-course was appropriate and informative.	44.4%	33.3%	18.5%	0.0%	3.7%	77.8%
2. The course was scheduled at a suitable time of year.	51.9%	29.6%	3.7%	14.8%	0.0%	81.5%
3. Overall, the course facilities and location were appropriate and satisfactory.	63.0%	14.8%	3.7%	14.8%	3.7%	77.8%
4. Overall, the course material was presented in a clear and organized manner.	40.7%	37.0%	11.1%	7.4%	3.7%	77.8%
5. Overall, the instructors were effective and responded to questions in an informative, appropriate and satisfactory manner.	59.3%	18.5%	18.5%	3.7%	0.0%	77.8%
6. Overall, the hangouts for discussion groups and case studies were clear and useful.	44.4%	33.3%	11.1%	11.1%	0.0%	77.8%
7. Overall, the course was informative and valuable.	51.9%	33.3%	7.4%	7.4%	0.0%	85.2%
8. Was the course above or below your current knowledge level?	Below 3.7%		Just right 59.3%		Above 37.0%	
9. Would you recommend this session to another colleague? (Yes/ No)	100% Yes					
10. In what ways could this course have been improved to better suit your needs?	48.2% Better					
11. Other comments.	Most comments were thankful					

Most respondents (77.8%) reported that the course was appropriate and informative, 81.5% indicated that it was scheduled at a suitable time of year, 77.8% agreed that the facilities and location were appropriate and satisfactory, and 77.8% said that the course material was clear and organized.

Moreover, 77.8% found that the instructors were effective and responded to questions in an informative, appropriate, and satisfactory manner, and 77.8% agreed that the handouts for discussion groups and case studies were clear and useful.

Finally, 85.2% of the participants found that the course was valuable. A third of the participants (37.0%) felt that the course was pitched above their current knowledge level, 59.3% that the content was at the right level while 3.7% found it below their current knowledge level.

One year after the course, thirteen of the 41 course participants (31.7%) responded to the post-event questionnaire to assess their satisfaction with the course and with retention of the knowledge and skills they gained. Eight (61.5%) indicated that it was the first time they had attended a course on this topic; all of them agreed that it was beneficial and that they still retained the knowledge and skills 1 year after the course. Seven participants (53.8%) indicated that they applied the principles learned from the course while 30.8% had participated in chemical response tasks (e.g. triage, treatment decontamination) after they completed the course.

## Discussion

Regardless of the healthcare professional’s background, healthcare providers who are likely to be involved in a disaster health response should acquire the basic knowledge and skills for identifying and detecting potential hazards and have the skills to establish preventive safety measures that will trigger a high-level response before CBRN TEAMs are deployed [ 37].

### Foundation level using a multi-disciplinary, blended-learning approach

*This foundation level* accredited competency based multi-disciplinary major chemical incident course was developed to provide and empower attendees with the knowledge required to respond to such events. It was implemented by the Higher Education Centre at King Fahad Security College (KFSC). As in other disaster health training courses, the assessment of knowledge gained showed no significant difference between health professionals (*P* > 0.05). This is likely due to the fact that the course was set at a foundation level (basic knowledge and skills for all healthcare providers in different professions) for healthcare providers, based on World Association for Disaster and Emergency Medicine (WADEM) and other international education frameworks. However, the literature confirms that the quality of care and assistance delivered during a disaster improves with inter-professional education [14, 27, 32, 35, 38, 42, 43].

#### Teaching strategy and approach

Responding to a major incident requires a unique approach, in addition to educational and training strategies that differ from daily emergency operations. The capability to function successfully under unsafe environments mandates repeated and specific training [37]. Unfortunately, despite the increasing number of CBRN threats, training initiatives over the recent years continue to follow traditional teaching approaches. Most of the available basic CBRN programs are lecture based, lacking simulation enhanced training exercises. Usually only advanced HAZMAT teams receive training programs that include hands-on exercises and simulation-based training [44].

*Cross-disciplinary approach* was used for medical and disaster health education to demonstrate the problems from a multiple disciplinary perspective [32, 45]. This approach was able to integrate across fields to apply knowledge in different contexts. Mixed experience sessions were created in the safety and protection domain by instructors from different fields [disaster medicine, toxicology, civil defence, occupational safety and health administration (OSHA)] who brought their perspective and expertise with regards to safety and protection measures.

After the different training sessions, the participants had an integrated perspective of the safety and protection measures that allowed them to fit their learning competencies into a larger knowledge context as observed in the tabletop exercise for the simulated organophosphate release and functional drill in emergency department for self-evacuated victims.

All critical aspects, like donning and doffing of PPE Level C, cross-disciplinary communication, triage and lifesaving procedures, mass decontamination, radio and verbal communication, command and control were discussed and practiced [37].

CBRNE incidents are unusual circumstances, using this approach allowed addressing of one major knowledge gap, which is multicausality scene safety/organization and functioning while in PPE. The main challenges and struggles observed were unfamiliarity with PPE equipment, communication while in PPE and command/control. These areas need to be highlighted and given additional time for practice in future courses. The health impacts of long-term PPE use (heat exhaustion) and effective communication with victims are other areas that need to be addressed.

The course was designed to be implemented over 5 days to allow for ample time to cover all topics to enhance the participants’ knowledge and skills in addition to the hands-on sessions such as decontamination, PPE use and triage which are only observed in courses with longer duration [3, 46–49].

#### Blended learning strategy

Blended learning is one of the most effective teaching methods in disaster medicine, via multiple teaching strategies it provides the opportunity to teach broad aspects of knowledge and skills. This blended approach is chosen for many CBRN courses [3, 27, 48, 50, 51]. A gradual progression of different educational strategies from the traditional educational lecture format to a complex enhanced functional drill was used to help create an effective learning environment that suits the different objectives of the course as well as the learning style of learners. This was reflected by the participants’ perceived motivation and high satisfaction scores on the feedback form. Using this approach was not only helpful for individual training, but also facilitated the broader understanding of their role and contributions towards interdisciplinary teamwork. From using this teaching method, it would be recommended to integrate different teaching strategies per day vs per course (daily blending vs course blending). As noticed having 3 days of lectures followed by the practical sessions at the end was less enjoyable and effected participants attendance. In future courses practical sessions will be implemented daily with the lectures and the simulation enhance drill will remain at the end despite the logistical challenges that might exist.

#### Simulation enhanced training

Simulation-based education has been shown to be a realistic and effective approach to prepare responders for these events, it increases the learners’ knowledge, enhances self-confidence and refines clinical skills when combined with deliberate practice and feedback [47, 52, 53]. The learners were involved in repetitive performance sessions to practice the skills required in PPE and decontamination, combined with decision making in triage to achieve competency mastery [48]. These key skills were used in their real-life practice with 53.8% of the participants indicating that they applied the principles learned from the course and 30.8% participating in chemical response tasks (e.g. triage, treatment decontamination) after they completed the course. During both the tabletop and functional exercise the learners were presented with realistic levels of complexities. Realistic scenarios can be a successful method for performance-level learning that requires learners to experience how specific disasters might progress over time [3]. Cross disciplinary communication was emphasized in both scenarios and radio communication was also used while wearing PPE and during all Multi-Sectorial Rescue Chain (MSRC) exercises, Standardized patients were used for mass decontamination and the functional drill to facilitate the training and practice of critical skills [3]. It is recommended that manikins are utilized in future courses to enable practicing lifesaving interventions while wearing PPE (e.g. endotracheal intubation, needle decompression) taking into consideration the profession of learners involved as these skills are considered higher level competencies, beyond the scope of this level. Victim cards when used in decontamination triage and the tabletop exercise, enhanced communication and discussion among learners enabling them to make informed decisions about the victim care management [54].

### Domain knowledge assessment

The main training goals for this course were to “ensure healthcare providers achieved the essential competencies (knowledge, skill, attitude) for implementation of the crucial mitigation and preventive safety measures and deliver basic medical care at all levels of the Multi-Sectorial Rescue Chain during mass chemical exposure [31, 30, 55]. These goals were achieved using performance objectives covered by the four knowledge domains. In this study a pre- and post-course test were conducted to evaluate the learners’ knowledge acquisition during the course. Debriefing sessions were also used to evaluate team performances.

The overall score on the pre-test (for all domains) was 46.3% which increased to 69.47% on the post-test. All the threat identification domain (Domain 1) items showed remarkably increased correct responses by the participants after the course sessions and this domain is the subject area which is most included in previous training courses [56]. Chemical incidents usually require involvement of toxicology and poisoning centres to provide real time advice and support for healthcare providers. About 33% of this domain tested the knowledge regarding the role of the poison centre, the use of a communication check list with advice and instructions from clinical toxicologists. A multidisciplinary and coordinated response is required to alleviate the mortality and morbidity among cases and conserve the healthcare system as well as the community [1, 27, 57]. Knowledge of the physical and chemical properties of the chemical substances were also tested in this domain using around 66% of the items to understand the concept of nature of chemical substance (gas, liquid) and its effect on mitigation and preventive measures during the response phase [24, 25, 27].

Since major chemical incidents are high impact, low frequency events, their health effects are unfamiliar to healthcare providers which in turn emphasizes the need for training programs aimed at medical respondents [3, 27]. To address the knowledge gap in this subject area, the course curriculum introduced the domain on the health effects of major chemical incidents (Domain 2). The results of the post-course test were significantly higher than the pre-course test for this domain.

In the medical response to major chemical incident domain (Domain 3), the subject areas were considered key components for a comprehensive disaster preparedness framework for both the pre-hospital and the in-hospital tiers of the medical chain [27]. The safety and protection measures are integrated along the whole pathway of the rescue chain. Deficiencies in the incident scene and providers safety can lead to both secondary contamination for the responders and definitive care hospital.

Blended learning in CBRN curriculum with lectures and simulation is required for skill acquisition [51], about 60% of the course was covered by interactive lectures and 40% by practice (hand on sessions, simulation enhance training).

### Team and individual performance evaluation

Team and individual performances were evaluated by instructors/observers and recorded notes according to performance objectives outlined for each hands-on session or simulation enhanced exercise. During the hands-on sessions, such as PPE donning and doffing, mistakes in skills performance were corrected immediately by instructors, and then the participants were asked to repeat the skill to achieve the competency level required. Checklists were used in decontamination, PPE, communication with the toxicology and poisoning centres for both hands-on sessions and simulation enhanced exercises to ensure that the required details were communicated. CBRN readiness checklists helped the instructors determine what additional training was required [37]. Team goals, organization, and coordination were areas that participants found challenging. Additional emphasis was given on these aspects during the simulation sessions because these have been demonstrated to increase team performance. Significant increases in knowledge and skills were observed after the hands-on sessions as demonstrated by other studies [3], and were apparent in the tabletop exercise and functional drill.

Debriefing was used after the simulation enhanced sessions (tabletop exercise, functional drill) to clarify any confusion and summarize and evaluate the team actions and performance according to the exercise objectives and the course required competencies. These included, but were not limited to, team performance, leadership, cooperation, communication, calls for additional resources, emergency declaration, medical triage and treatment. The debriefing sessions were considered a self-evaluation process with instructors serving as facilitators [47].

### Participant feedback

In disaster health (including CBRN threats), education and training are quite different from other medical fields due to the patterns of disasters and the different factors that affect the response outcome. Consequently, no single method can assess the value of training programs and multiple methods can be used [14, 32]. The course evaluation process is vital to demonstrate the gaps and the needs for further improvement in the future and how to incorporate aims and objectives of healthcare organizations and individuals who participated [3, 14, 32, 58]. This course was evaluated by different tools for multiple components, including pre and post-tests, instructor and observer reflection, debriefing sessions and participant feedback. All participants indicated their interest and agreed that they met their personal goals. About 37% of the participants indicated that the course was above their knowledge level and this could be explained by the lack of disaster health awareness, inadequate education, training and lack of drills around disaster health in the different health sectors [21–23, 41]. In addition, many participants had just attended, 2 weeks prior, their first general disaster health awareness course, and felt that the field was a very new subject for them.

Major chemical incidents are complex and require a combination of highly precise skills and education. It is essential that the participants retain their knowledge and skills gained in a course over time [37]. In these cases, the technical skill-based components appear to decline at a faster rate than knowledge. This faster decrease in skills retention could have a critical impact in major chemical events, in which the use of PPE is essential and decreased competence may lead to hesitation and reduced performance in a contaminated area, in addition to knowledge defects [59]. The blended learning delivery in disaster health training can enhance confidence in learners’ knowledge for at least 6 months [58, 60]. In the post-course survey, all participants agreed that they still retained the knowledge and skills 1 year after the course. The confidence in participants is not necessarily reflected in the maintenance of competence. Short, refresher courses are required annually if healthcare providers are not exposed to critical events on a regular basis as their knowledge and skills in responding to such events can be reduced 6–12 months after their initial training [61].

The results extrapolated from the multiple evaluation methods in this study can be used to guide the national educational framework for CBRN threats.

## Limitations

One of the main obstacles in this course is the language barrier as many paramedics prefer to use Arabic for theoretical and practice sessions according to a WADEM survey in 2004 on the current status of disaster medicine education. In the survey, about 29% of the participants suggested separating domestic courses from international courses and to teach the courses in the native language with translation of the course material [42]. Other obstacles include the lack of commitment and poor attendance of the participants to some lectures especially during the third day which affected the results of knowledge gain in some domains. The psychological impacts resulting from CBRN threats need to be emphasized more in this course, the lack of knowledge among healthcare providers on how to successfully manage these impacts has potential wide-reaching effects and behavioural consequences, which in turn have an influence on morbidity and mortality rates [62, 63].

Skill retention was measured subjectively by self-reflection, it would be ideal to repeat a simulation session to evaluate retention of skills. Given that this was a pilot study with no financial support, it was logistically not feasible. In the future once similar courses are implemented, a refresher course within 6–12 months to measure and re-emphasize skill retention is recommended.

## Conclusion

Multi-disciplinary competency-based blended learning education programs in major chemical incidents may increase healthcare providers skills and knowledge that is important for improvement of the level of national preparedness and subsequent staff availability. This foundation level course is for front line healthcare providers who will likely be involved in a major incident or disaster and will make a crucial difference in reducing the health impact of these incidents. These front-line healthcare providers can support community health groups in disaster risk reduction strategies in collaboration with other sectors (HAZMAT teams in civil defence, poisoning centres, regional command centres with the Ministry of Health).

## Supplementary Information

**Additional file 1.**

**Additional file 2.**

## Data Availability

The datasets generated during and/or analysed during the current study are available from the corresponding author on reasonable request.
