# Supplementary material for: Assessment of the effectiveness of a course in major chemical incidents for front line health care providers: a pilot study from Saudi Arabia
Source: BMC Med Educ. 2022 May 9;22:350. doi: 10.1186/s12909-022-03427-2 (PMC9082960; doi:10.1186/s12909-022-03427-2)
Supplement: Supplementary file 1 — Additional file 1. [file 12909_2022_3427_MOESM1_ESM.docx]

**Additional Materials**

**Table A1 – Core competencies and sub-competencies in each domain**

| **Domain** | **Core Competency** | **Sub-Competencies** | **Teaching Methods** |
| --- | --- | --- | --- |
| 1-Threat identification | Demonstrate knowledge of chemical hazards identification from different perspective areas (terms and characteristics, authoritative source of information) | 1. Define the key terms for chemical major incident 2. Recognize and identify different Chemical Agents scenarios 3. Understand the factors influencing the effects of chemical incidents 4. Illustrate the role of poisoning center during chemical incidents 5. Understand the operation activities of the poison control center in case of a disaster 6. List the recommendations for national plan related to toxicology and disaster preparedness | **Interactive presentation**  -Overview of chemical major incidents  -Role of toxicology centre in chemical incidents  **Simulation enhanced training sessions**  -Table top exercise for organophosphate scenario  - Drill for self-evacuation to hospital |
| 2-Health effect of chemical agent | Demonstrate knowledge of health impact consequence due to exposure to chemical /chemical warfare major incidents | 1. Understand the proper action steps for suspected chemical incidents 2. Understand and list the ABC’s of the management of chemical incidents events 3. List and identify the common toxidrome in chemical warfare incidents 4. Recognize the patterns of sign and symptoms observed in mass chemical exposure 5. Understand the main principles for treatment of specific chemical agents | **Interactive presentation**  - chemical toxidromes potential health effect, action steps for hazard mitigation |
| 3-Response to chemical major incidents | Demonstrate knowledge of principles for chemical incident response | **Pre Hospital:**   1. Understand the standard operations procedures for conformation and identification processes at incident scenes 2. Explain the communication and organization strategies with other agents, e.g. civil defense, toxicology center 3. Determine and understand the sequence of measures at scene (triage, primary assessment, treatment, transportation) 4. Apply the safety measures required for safety of rescue workers and population 5. Understand the general rules for transportation of victims of chemical incidents   **Hospital (mainly ER)**   1. List the top priority for hospital response and management (safety, scene contamination) 2. Understand the SOP for alert and notification processes, activation of the plan (including activation of HICS) 3. Understand the actions required for preparing the hospital for receiving the victims (HAZMAT team and other staff, decontamination facility and other medical equipment and antidotes, space including ICU BEDS) 4. Describe patient flow through emergency department during mass chemical exposure 5. Understand the general principles for victim management (primary and secondary decontamination, primary triage, immediate RX, secondary triage, toxic trauma treatment, admission) 6. Apply the principle of primary and secondary triage in ED.   **Toxic Trauma Treatment**  management of most common chemical agent (Intentional-No intentional) in mass chemical exposure incident   1. Understand the general approach to toxic trauma management in all rescue chain during mass chemical warfare incident (evacuation, persistence information, primary examination, toxic trauma treatment, antidote, observation and admission, discharge criteria). | **Interactive presentation**  Mass chemical pre-hospital response  **Interactive presentation**  Hospital response and management  **Interactive presentation**  Toxic trauma management for specific chemical warfare  **Simulation enhanced training sessions**  -Table top exercise for organophosphate scenario  - Drill for self-evacuation to hospital |
| 4- Basic Concept in Protection and Safety | Demonstrate proficiency of minimum protection measures and safety principles for chemical incidents | 1. Understand and list the types of PPE and decontamination facility used in CBRN threats, apply the most common type of PPE used in mass chemical incidents for healthcare providers in cold and warm zones 2. Apply the procedure for decontamination triage in ambulatory and non-ambulatory victims 3. List and understand the types of decontamination 4. Demonstrate the principles for mass decontamination 5. Understand important aspects during child decontamination 6. Understand decontamination process in challenging weather conditions | -Personal protective equipment levels and types  -General principles in mass decontamination  **Hands-on sessions and simulation enhanced training sessions**   - Types and levels of PPE and decontamination facility - Hands-on training for PPE Level C - Wound decontamination and antidote - Triage decontamination - Table top exercise for organophosphate scenario - Drill for self-evacuation to hospital |

**Stages of Course Development**

**Stage 1: The need of training for non-conventional incidents for health care providers in Saudi Arabia**

A comprehensive literature review was done to highlight the need for competency-based education in disaster health including Chemical, biological, radiological and nuclear (CBRN) subjects in Saudi Arabia. There were no published reports assessing and evaluating a specific course for CBRN subjects, only one article for prehospital major incident response introduction course was identified that distinguished and demonstrated the use of different levels (mainly levels C and D) of Personal Protective Equipment (PPE) that is part of the safety and protection domain.^1^

**Stage 2: Preference Major Chemical Incident Course vs. Other Unconventional Threat (Biological, Nuclear, Radiological Threats)**

The subject matter experts after multiple brainstorming sessions identified which CBRN threats are more likely in the Middle East area and analysed the characteristics of the possible CBRN threats that can specifically affect the Saudi community.^2^ Many challenges and circumstances were found including political unrest in the Middle East ^1^ with increasing incidents of chemical attacks against civilians in Syria in 2013 and the growing risk from chemical industry accidents with an unsafe industry environment for the workers in Saudi Arabia. This highlighted lack of hospitals preparedness to chemical incidents and the escalating need of healthcare providers with adequate training in major chemical incidents. ^3-12^ After reviewing all the above mentioned challenges and the sponsor logistic capacity, the course experts identified the chemical threats compared to other non-conventional threats.

**Stage 3: Delineation of the foundation level for chemical major incidents according to participants' role and tasks**

Most Disaster Health Education frameworks were designed according to international standers and local context.^1, 14^ The majority of previous frameworks composed of different levels, each level has competencies sets, and the competencies sets for the each higher level increase in complexity according to subject areas, professionality, organizations, and their roles during disaster response.^14-18^ Although it would be unfeasible to provide all healthcare providers (responders and receivers) with advanced level CBRN training, the experts agreed that all front-line healthcare providers should receive at least the foundation level training for CBRN threats. This level would include the fundamentals of safety for hazard identification, knowledge of health effects, appropriate PPE, decontamination process.^2,19,20^ This foundation level is for advanced knowledge delivery of competency sets for basic knowledge and essential operational skills in major chemical incidents. This level allowed healthcare workers to do initial assessments, identify the chemical hazards, assist the HAZMAT team in warm zones with documentation and registration, ambulatory decontamination, medical triage post decontamination, and exchange information with the poison centers.^2,20^

The qualifications involved in this course focused more on basic knowledge and essential skills for identification of the hazards, main signs, symptoms, the role of the poisoning center and communication/coordination principles. Higher level training could follow as seen in Table A2 below.

**Stage 4: Competencies development**

The core competencies set for CBRN threat including mass chemical exposure education were identified after a comprehensive literature review. Four domains were determined according to participants' role and tasks and six levels of proficiency (Bloom’s Education Taxonomy) and were used to establish the core competencies and sub-competencies for the foundation level in major chemical incident. ^2, 17, 18, 21, 22^

The Foundation level course was composed of four main domains and core competencies and 28 sub-competencies (see Table A1).

A Blended learning technique was used (interactive presentation, table top exercise, drill, experiential/ hand on exercise) to deliver knowledge and skills and promote higher cognitive engagement.^1, 23^ Each domain was covered by a theoretical and operation part**.**

**The four main knowledge domains**

1. **Threat Identification Domain**

The threat identification domain was composed of one core competency and 6 sub-competencies. The domain introduced three topic areas: different chemical agents’ experiences and scenarios from the past, factors influencing the effects of chemical incidents, and the role of the poisoning centre during a major chemical incident. An interactive presentation was the teaching method used. The chemical threat identification domain was approached and illustrated from different perspectives (terms, previous experiences characteristics, authoritative source of information). An overview to mass chemical exposure incidents lectures, different terms and definitions were explained with previous experiences from intentional and non-intentional mass chemical exposures (e.g. German and French Troop, Troops Bhopal Gas Tragedy).

Factors Influencing the Effects of Chemical substances like Persistency, Effectiveness, Physical properties and route of exposure were presented and cogitated as key properties of chemical agents the learners understood the risk of secondary Contamination for persistence vs. non-persistence agents according to the form of the substance (gas, liquid, solid) and its effect on the health of the victims exposed to hazards for considerable periods of time.

Regarding the Physical Properties, it explained how the percentage value for the vapour density in the chemical agent can keep the chemical gas heavier or lighter than air and if the gas will rise or sink in the incident area which is an important factor for first responders to understand in order to take the protective measures and identify the nature of the chemical agent.

The last topic covered in this domain emphasized the poison center roles and activities for mass chemical exposures. The learners needed to understand the main roles of the center to support health care providers in threat identification and management. Rescue management plans for the poisoning center play a significant role in the risk assessment model. The assessment starts when the health care providers give the information about the type of accident, potentially hazardous substance involved, their amount, number of people exposed to health problems. From previous data, the center can identify the hazards, assess the dose-response to determine the health problems for the exposures, and exposure assessment for people exposed to the pollutant during specific times.

On the basis of this analysis, the toxicology center provides the health providers with basic instructions about the potential risk of poisoning to the exposed, first responder’s protection, decontamination procedures, the triage recommendation, and the basic information about treatment including administration of antidotes.

1. **Health Effect of Chemical Agent Domain**

The health effect of the chemical agent domain was composed of one core competency and five sub-competencies. The domain introduced the steps needed for a health care provider to follow when they suspected the incident (intentional and non-intentional) is chemical in nature regardless of the type of the material.

Health care providers need to receive information about the nature of the material, and its effect which can help them to take protective actions that can support them in managing the incident. Also understand the potential health impacts for suspected chemical hazards and its consequences. Multiple resources were illustrated to the learners like (master safety sheet, online documentation) for knowing the clinical findings. The mechanism of action with signs and symptoms for main chemical agent were illustrated. The Differentiate between biological and chemical threats aspect was also explained.

1. **Response to Major Chemical Incident Domain**

The response to the major chemical incident domain composed of one core competency with five sub-competencies for pre-hospital response and 6 sub competencies for hospital response and one sub competency for toxic trauma treatment.

The pre-hospital response setting video for Tokyo Sarin subway in 1997 was presented for seven minutes followed by discussion of the main issues that happened during the response phase by EMS team and first responders. The vital tactical activates like integration and cooperation with concerned organizations, regional mediator by poison information centers, and establishment of a multidirectional communication system) that is recommended by health authorities following the incident were discussed with learners.

The prehospital response lecture discussed the important operations required for scene management. Like conformation and identification process at incident scene. The learner was informed about signs and clues that can lead to possibility of hazards substance release. For rapid and accurate recognition and detection, two check lists were presented. One for additional data that required by receiving chemical incident call and the other for communication with clinical toxicologist.

The second section explained the general role for the safe access to the incident scene with standards required for site organization by establishing the restricted areas hot, warm and cold zones. Special considerations were introduced for treatment of the victims with sequences of measures like hot zone evacuation, registration, undressing, mass decontamination, spot decontamination with wound dressing, primary triage, antidote administration, secondary triage, and transportation to hospital.

The indications for life-saving treatments for the patient before during and after the decontamination and the precautions required to avoid secondary contamination were also highlighted.

**Hospital response lecture**. The topic area mainly focused on hospital organizational level (procedures) and technological level (e.g. decontamination, communication, security, medical triage, treatment). To ensure effective detection and verification for chemical events, several sources of notification and alert were illustrated in this part. For integration and cooperation of concerned organizations and interdepartmental coordination in the same the hospital, the hospital incident command system (HICS) activation positions with action cards related to mass chemical exposure were explained in this lecture.

To enhance the hospital's ability to accommodate a sudden transient influx of patients. The hospital preparation from different perspectives (e.g. decontamination facility space, hospital stockpiles, ICU beds, department beds) with patient flow through the emergency department was introduced to the learners.

The last part for the response domain is a toxic trauma treatment of the most common poisonous injury. This part offered the basic knowledge for EMS and hospital receivers’ teams and presented the essential information in a simple approach. The information presented in the lecture considered as starting point for health care providers and list crucial aspects required for the management of the victims for both pre-hospital and hospital setting (e.g., evacuation in pre-hospital, the persistence of substance, type of PPE, preliminary examination, toxic trauma treatment, antidote, observation, admission/discharge).

**4. The Basic Concepts in Protection and Safety Domain**

The basic concept in protection and safety is composed of one Core Competencies and seven sub-competencies. Because the risks in the chemical incident for the rescuer's health or life might be high during transportation, receiving, or treating the victims who may have been contaminated or made ill from the incident. The rationale, function, type, limitation of personal protractile equipment (PPE), and decontamination process were presented in this domain. The basic requirements and type of decontaminations including mass decontamination, decontamination of the children, and decontamination in cold weather) were presented in this domain.

In the chemical incidents, the number of apparent victims may exceed the capability for successful rescue/evacuation, decontamination, and treatment of the victims, Decontamination triage was introduced in this part to illustrate to the learner how to make decisions for the patients who need decontamination first, Patients who needs urgent lifesaving before decontamination , the problem of secondary contamination has been highlighted in the emergency departments that may have to receive casualties who have been incompletely decontaminated at the incident site or those who have self-evacuated.

**The Practical Components of the Course**

On day 4 and 5, small group sessions and simulation-based exercises were conducted. In the small group sessions, the learners rotate into four groups (9 to 10 individuals). The sessions were composed **of an awareness section and hands-on sections** for emergency response skills that were presented in the theory section.

The awareness session introduces the hazmat detection and management vehicle, the chemical decontamination tent, and the decontamination car in addition to the types of personal protective equipment. The hazardous materials team from civil defence provided the basic information and demonstration for the aforementioned equipment.

The hands-on sessions focus on the skills training for different operational aspects as follows:

1. Personal protective equipment (PPE) Level C techniques and order for donning and doffing. Errors in skill performance were corrected immediately and then were repeated to achieve the requested skills.
2. Ambulatory and non-ambulatory decontamination including wound decontamination in different area; eye, chest and arm.
3. Triage decontamination by prepared 10 cases with clinical information (sign and symptoms and parameter of each patient) printed on a white card for an organophosphate agent. Each group was asked to follow the flow chart provided to them and to categorize each patient and their priority for decontamination. After 15 minutes, the instructor disused the triage category for each patient and corrected the incorrect category for them.

In the afternoon session on the fourth day, a tabletop simulation exercise involving a nerve agent release as a terrorist attack at the Annual Conference for Police Academies resulting in injury of 30 people. The main objectives were to exercise the coordination and communication between first responders and other response agencies including the poison centre, safety measures required for health care providers in chemical incidents, victims’ management (Triage, Antidote, decontamination, and Transportation) and to emphasize the role of front-line Health care providers in such events.

Multidisciplinary team-building concepts were practiced by having participants from other agents (HAZMAT team - civil defence, law enforcement team, and poisoning center team). The participants covered dispatch and regional command center and prehospital scenes. The exercise was conducted in the big conference room and the area was divided into different stations. Each station in the pre-hospital scene was represented including the scene station, decontamination station, and primary and secondary triage. The other side of the conference room was designated as the poisoning center, regional and dispatch center with tables and chairs and a whiteboard in each station.

At each station, two instructors observed the management of the victims and evaluated the actions taken by team members. The facilitators were assigned for hospital command groups, the callers from the incident scenes asked for help and records to dispatch centre.

In each case, the history of the patient (signs and symptoms and physiological parameters) was written on small magnetic cards with a code number. The first ambulance arrived and took responsibility for command of the scene. When the other team arrived, the incident commander distributed them to the scene stations for triage decontamination, primary and secondary medical triage and transportation. The learners provided to each station were provided with small colored stickers for triage and samples with logos for basic life support and antidote treatment.

Whenever the learners needed more information about the agent recognized, its health impact, level of protection, antidote administration, and dose, they could contact the poisoning center. Operation, command, communications and control principles were stressed as participants used a two-way radio to communicate with the relevant organization. The exercise was repeated twice with 20 participants each time to give opportunities for each team member to apply the standard operation procedures for the response. After the second group finished, a debriefing of the session was conducted for all participants.

On the last day, the drill for self-evacuation victims to the emergency department due to an explosion of ethylene oxide storage tanks in a factory was conducted. The main objectives for this drill were to practice the emergency department's immediate actions while receiving the self-evacuation patients including safety precautions measures required for the department staff, patients, and the standard operating procedures for patient management (e.g. triage, lifesaving procedures, decontamination, and toxic treatment). This also included practicing communication with the toxicology center for identification of chemical material, treatments, PPE, and decontamination required.

The instructors prepared a big tent for receiving the victims as an emergency department entrance, reception area, and triage for victims. A mobile decontamination (for ambulatory and non-ambulatory victims) car was prepared 200 m away from the emergency department area, another area closed from decontamination care was designated for the treatment of patients post-decontamination. The participants contributed as observers and players. Twelve standardized patients were used in this scenario and two-way radios were used to facilitate the communication of the players with the regional command center and toxicology center. Debriefing was conducted post drill. The small group session covered mainly; the basic concept in protection and safety domain while tabletop exercise and drill covered all domains. The debriefing session for both the tabletop exercise and the drill was conducted by simulation educators, whom where observers during the exercise using checklists. The aim for the debriefing time was to approximate double the actual simulation drill as this is believed where most of the learning occurs. One of the standard tools of debriefing “Plus delta” was used to identify areas that went well and areas that need improvement.

**Outcome Measures**

Three stages of Kirkpatrick’s Four Model were used to develop cognitive assessment, feedback evaluation and post event questionnaire forms.

1. **The Cognitive assessments level 2 (knowledge, skills and attitude)**:

The learners completed a 25 multiple-choice question examination before and after the course. Content and difficulty were same for all pre-course and post-course assessments. The instructors were asked to prepare questions relevant to their subjects. Each 30-minute test consisted of 25 multiple choice questions with only one correct answer. One point was given for a correct answer, zero for a wrong answer. Four instructors reviewed all items for both tests for face validity. For individual skills assessment, correction of the mistakes was done immediately and then skills were repeated in decontamination, donning, and doffing of the PPE Level C until the required standard was achieved. For the team performance in the decontamination triage session and simulation-based exercise (table top exercise and the drill), the observation was followed by a debriefing sessions and used as self-evaluation for reviewing the actions and measures that were taken for response management; (e.g. command control, communication and coordination, emergency declaration, decontamination and triage, selection the levels of PPE, Information management, recognition and medical care for chemical substances).

1. **Feedback (learner stratification)**

An 11-item evaluation questionnaire was used to collect feedback from participants at the end of the course. The feedback included evaluation of the effectiveness of the course and suggestions on how the course might be improved. The survey took about 10 minutes to complete. The first seven questions (mostly Likert scale) asked about course level knowledge, material, time and location. The last four questions involved two open-ended and two select one of the answer in the small boxes.

**c. Measurement of Behaviour Level Post-Course**

One year after the course, a follow-up survey was sent to the participants by email. Those who **did not respond to the email were contacted by phone** whenever possible. The questionnaire is presented below.

**Table A2: Post-Event Questionnaire**

| No. | Questions |
| --- | --- |
| 1 | Was this the first course you attended in this topic? (Yes/No) |
| 2 | Was this course Beneficial to you? (Yes/No) |
| 3 | Did you obtain the knowledge and skills needed from this course? (Yes/No) |
| 4 | Did you apply any principles learned from this course since taking it? (Yes/No) |
| 5 | Did you participate in any chemical response tasks (e.g. triage, treatment decontamination) after you completed this course? (Yes/No)  If you answered YES, how effective was this course in preparing you for the Chemical Incident Response? (Strongly agree (1) / Strongly disagree (5)). |

**References**

1. Bajow, N., AlAssaf, W. and Cluntun, A. Course in Prehospital Major Incidents Management for Health Care Providers in Saud Arabia. Prehospital and Disaster Medicine.2018; 33(6), pp.587-595.
2. Djalali, A., Della Corte, F., Segond, F., Metzger, M., Gabilly, L., Grieger, F., Larrucea, X., Violi, C., Lopez, C., Arnod-Prin, P. and Ingrassia, P. TIER competency-based training course for the first receivers of CBRN casualties. European Journal of Emergency Medicine.2017; 24(5), pp.371-376.
3. Alamri YA. Rains and floods in Saudi Arabia. Crying of the sky or of the people? Saudi Med J. 2011;32(3):311-313.
4. Brooks, J., Erickson, T., Kayden, S., Ruiz, R., Wilkinson, S. and Burkle, F. Responding to chemical weapons violations in Syria: legal, health, and humanitarian recommendations. Conflict and Health. 2018; 1(1).
5. Hakeem O, Jabri S. Adverse birth outcomes in women exposed to Syrian chemical attack. LancetGlob Health. 2015; 3(4):e196.
6. Emad Abukhashabah, E. Causes of Occupational Accidents and Injuries in Construction Industry in Jeddah City. Journal of King Abdulaziz University - Meteorology, Environment and Arid Land Agriculture Sciences. 2019; 28(1), pp.105-116.
7. Alsubiaee, K., Alsharani, M., Alazmi, A. and Alsadoon, R., 2017. Implementation of Safety Standards in Saudi Arabian Scientific Laboratories: An Empirical Study. Journal of Arthritis. 2017; 06(04).
8. Makki, A. and Mosly, I.Determinants for Safety Climate Evaluation of Construction Industry Sites in Saudi Arabia. International Journal of Environmental Research and Public Health.2020; 17(21), p.8225.
9. Al-Shareef AS, Alsulimani LK, Bojan HM, Masri TM, Grimes JO, Molloy MS, et al. Evaluation of Hospitals’ Disaster Preparedness Plans in the Holy City of Makkah (Mecca): A Cross-SectionalObservation Study. Prehospital Disaster Med. 2017;32(1):33–45.
10. Bin Shalhoub AA, Khan AA, Alaska YA. Evaluation of disaster preparedness for mass casualty incidents in private hospitals in Central Saudi Arabia. Saudi Med J. 2017;38(3):302–6.
11. Shammah, A., 2018. Preparedness Assessment for Disaster Management Among Dhahran Al Janoub General Hospital Staff During Hazm Storm Support. International Journal of Community and Family Medicine. 2015; 1436 3(1).
12. Khan. A, Alahamari. A. Chemical, biological, radiological, and nuclear preparedness of public hospitals in Riyadh. Nov. 2020. Available at: <https://www.researchgate.net/publication/346882092_Chemical_biological_radiological_and_nuclear_preparedness_of_public_hospitals_in_Riyadh>
13. The Education Committee Working Group of the World Association for Emergency and Disaster Medicine: International standards and guidelines on education and training for the multi-disciplinary health response, an issues paper. Prehosp Disaster Med 2004; 19(3):186-187.
14. The Education Committee Working Group of the World Association for Emergency and Disaster Medicine: International standards and guidelines on education and training for the multi-disciplinary health response, an issues paper. Prehosp Disaster Med 2004; 19(3):186-187.
15. Murray V, Clifford J, Seynaeve G, Fisher JM.Disaster health education and training: a pilot questionnaire to understand current status. Prehosp Disaster Med. 2006; 21(3):156-167
16. Walsh L, Subbarao I, Gebbie K, et al. Core competencies for disaster medicine and public health. Disaster Med Public Health Prep. 2012; 6(1):44-52.
17. Schultz CH, Koenig KL, Whiteside M, Murray R. Development of national standardized all-hazard disaster core competencies for acute care physicians, nurses, and EMS professionals. Ann Emerg Med. 2012;59(3):196-208.
18. Subbarao I, Lyznicki JM, Hsu EB, et al. A Consensus-based Educational Framework and Competency Set for the Discipline of Disaster Medicine and Public Health Preparedness. Disaster Med Public. 2008;2(1):57-68.
19. Fitz Gerald, G., Aitken, P., Arbon, P., Archer, F., Cooper, D., Leggat, P., Myers, C., Robertson, A., Tarrant, M. and Davis, E., 2010. A National Framework for Disaster Health Education in Australia. Prehospital and Disaster Medicine. 2010; 25(1), pp.4-11.
20. Kollek, D., Welsford, M. and Wanger, K., 2009. Chemical, biological, radiological and nuclear preparedness training for emergency medical services providers. CJEM. 2009; 11(04), pp.337-342
21. Adams NE. Bloom’s taxonomy of cognitive learning objectives. J Med Libr Assoc. 2015; 103(3):152-153.
22. Linney, A., George Kernohan, W. and Higginson, R. The identification of competencies for an NHS response to chemical, biological, radiological, nuclear and explosive (CBRNe) emergencies. International Emergency Nursing.2011; 19(2), pp.96-105.
23. Bajow, N., Alawad, Y. and Aloraifi, S.A Basic Course in Humanitarian Health Emergency and Relief: A Pilot Study from Saudi Arabia. Prehospital and Disaster Medicine.2019; 34(6), pp.580-587.
