# Supplementary material for: Assessment of the effectiveness of a course in major chemical incidents for front line health care providers: a pilot study from Saudi Arabia
Source: BMC Med Educ. 2022 May 9;22:350. doi: 10.1186/s12909-022-03427-2 (PMC9082960; doi:10.1186/s12909-022-03427-2)
Supplement: Supplementary file 2 — Additional file 2. [file 12909_2022_3427_MOESM2_ESM.docx]

**PROFESSIONALIZATION**The competency set for the next (higher) level are cross-referenced for each professional group followed by a competency set with more advanced knowledge and skills reflecting the operational, tactical /and strategic needs for health planning according to the national health care organizations (Table AM1).

**Table AM1:** The competency set levels according to target audiences, subject areas, and professionalization

| **LEVEL** | **DESCRIPTION** | **TARGET AUDIENCE** | **REQUIREMENTS** | **SUBJECT AREAS** | **DURATION** | **PROFESSIONALIZATION** | **ISSUED CERTIFICATE duration** |
| --- | --- | --- | --- | --- | --- | --- | --- |
| Basic / Foundation | Competency set delivers basic knowledge and essential skills for main subject areas in chemical major incidents  This level is a foundation for more advance /specific competencies (in other levels | All front-line health care providers (responders and receivers) who will likely be involve in a major chemical incident response | - Saudi Health Commission Registration - Attended Major Incident Response Course | - Identification of the hazards  -Role of the poisoning center  -Prehospital and hospital response  -General principle for decontamination  -Principles in selection and use of PPE | 5 days (if there’s tactical training) | - Medical triage (cold zone)  - Affect Communication and Integration with poisoning center and other agencies  - Registration, initial, clinical assessment and documentation of relevant patient information in warm and cold zone  - Mass ambulatory decontamination in the warm zone.  - Assessment of HAZMAT team (assess the entry team) | Valid for one year with a refreshment course within 6-12 months for both knowledge and technical operations  (Donning and doffing PPE) and decontamination processes.  **Note:**  Knowledge component can be delivered via an online Module. |
| Advance/  Professional | Competency set with more extensive knowledge and advance skills  and cross reference for each professional group (paramedic, nurses, physicians) | Health care providers who play a significant role in major chemical incidents or plays a role in the HAZMAT team | - Saudi Health Commission Registration - Holds a valid BASIC Major Chemical incident course certificate (covers theoretical and operations related to basic competency set) | - Advanced TOXIC treatment  - Life saving procedures during decontamination  - Decontamination triage  - Non ambulatory triage  - Administration of antidote  - Personal and equipment’s decontamination | 2-3 weeks | - Plays an affective role as a member in the HAZMAT team  - Provides lifesaving procedure before /during decontamination  - Personal and equipment decontamination  - Warm zone triage (for decontamination and medical triage)  - Set up decontamination facility  - Ability to give the proper antidote and titration. | Valid for one year with a refreshment course within 6-12 months mainly for technical operations. |
| Expert/  Specialization | Competence set highly specialized in integrated operation, strategy, and tactical planning. | Health care providers who are responsible for leading and preparing the health care institution plans and educational initiatives. | - Advanced course in Major Chemical incident or Major CBRN course  -3-years’ Experience in Major chemical incident or CBRN management  - Master’s degree/Fellowship in disaster medicine management. | Tactical, Operational and Strategy preparedness for health care organization. | 4-6 weeks | - Plays an affective role as a member in the Incident command team  - Leads the team in Major Chemical incidents  - Ability to prepare and update the Healthcare organization plans based on hazard vulnerability analysis (HVA) | Yearly refreshment course to review national policies and updates. |
